# ddRAD‐Seq reveals evolutionary insights into population differentiation and the cryptic phylogeography of *Hyporhamphus intermedius* in Mainland China

**DOI:** 10.1002/ece3.9053

**Published:** 2022-07-04

**Authors:** Gongpei Wang, Han Lai, Sheng Bi, Dingli Guo, Xiaopin Zhao, Xiaoli Chen, Shuang Liu, Xuange Liu, Yuqin Su, Huadong Yi, Guifeng Li

**Affiliations:** ^1^ Guangdong Province Key Laboratory for Aquatic Economic Animals State Key Laboratory of Biocontrol School of Life Sciences Sun Yat‐Sen University Southern Marine Science and Engineering Guangdong Laboratory (Zhuhai) Guangzhou China; ^2^ State Key Laboratory of Ophthalmology Zhongshan Ophthalmic Center Sun Yat‐Sen University Guangzhou China; ^3^ Guangdong Provincial Engineering Technology Research Center for Healthy Breeding of Important Economic Fish Guangzhou China

**Keywords:** ddRAD‐seq, heterogeneous environments, *Hyporhamphus intermedius*, phylogeography, population subdivision

## Abstract

Species differentiation and local adaptation in heterogeneous environments have attracted much attention, although little is known about the mechanisms involved. *Hyporhamphus intermedius* is an anadromous, brackish‐water halfbeak that is widely distributed in coastal areas and hyperdiverse freshwater systems in China, making it an interesting model for research on phylogeography and local adaptation. Here, 156 individuals were sampled at eight sites from heterogeneous aquatic habitats to examine environmental and genetic contributions to phenotypic divergence. Using double‐digest restriction‐site‐associated DNA sequencing (ddRAD‐Seq) in the specimens from the different watersheds, 5498 single nucleotide polymorphisms (SNPs) were found among populations, with obvious population differentiation. We find that present‐day Mainland China populations are structured into distinct genetic clusters stretching from southern and northern ancestries, mirroring geography. Following a transplant event in Plateau Lakes, there were virtually no variations of genetic diversity occurred in two populations, despite the fact two main splits were unveiled in the demographic history. Additionally, dorsal, and anal fin traits varied widely between the southern group and the others, which highlighted previously unrecognized lineages. We then explore genotype–phenotype‐environment associations and predict candidate loci. Subgroup ranges appeared to correspond to geographic regions with heterogeneous hydrological factors, indicating that these features are likely important drivers of diversification. Accordingly, we conclude that genetic and phenotypic polymorphism and a moderate amount of genetic differentiation occurred, which might be ascribed to population subdivision, and the impact of abiotic factors.

## Graphical abstract

Hyporhamphus intermedius is widely distributed in in Mainland China, making it an interesting model for research on phylogeography and local adaptation.ddRAD‐Seq reveals evolutionary insights into population differentiation and the cryptic phylogeography of Hyporhamphus intermedius in heterogeneous environments in Mainland China

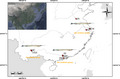

## 1 INTRODUCTION

Elucidating the origins of phenotypic diversity and the processes by which different modes of dispersal generate spatial patterns and the structure of genetic variation remains challenging for evolutionary biologists and ecologists (Avise, 2000; Saenz‐Agudelo et al., 2015). Several numbers of organisms occupy large geographic and very heterogeneous areas, which may shape the patterns of gene flow among populations across their range and drive population subdivision (Riginos & Liggins, 2013; Slatkin, 1987). Limited dispersal and low connectivity drive the fine‐scale population structure and may be more common than expected (Bluher et al., 2020; Chaichoompu et al., 2020). When different populations experience different environments, the accumulation of genomic divergence can modulate their underlying genomic architecture and drive phenotypic divergence, promoting local adaptation and novel natural lineages (George, 2000; Nosil et al., 2009; Via, 2001). These are of critical importance in population differentiation and speciation, which occurs due to the differential pressures of natural selection on populations in different environments resulting in allele frequency shifts; advantageous variation may accumulate over many generations to maximize fitness in a local environment, eventually forming distinct genetic and phenotypic traits (Andersen et al., 2011). The evolutionary forces driving genetic differentiation can be examined by comparing genomic information and environmental variables of populations (Sultan & Spencer, 2002; Wang et al., 2012). Local selective pressure can counter geographic and environmental gradients in latitude, and altitude. Genome–environmental association analysis can show selection at increasing geographic scales owing to larger environmental gradient differences (Fraser et al., 2011). Overall, discerning the relative contributions of geographic environmental factors and identifying factors such as natural selection, genetic drift, and dispersion that modify the patterns of genomic variation is critical when deducing the mechanisms by which organisms adapt to the local environment and initiate evolutionary diversification.

Fish are ideal organisms for studying local adaptive divergence and phenotypic plasticity. Given the complex geomorphic conditions in China, with dramatic climate changes at high altitude and latitude, the hyperdiverse freshwater systems (e.g., the Pearl and Yangtze Rivers and Plateau Lakes) show remarkably high levels of fish diversification and are prone to endemism. These independent areas separated by long distances probably also facilitated the differentiation of fishes and formation of the genetic landscape (Stoddart et al., 1978). Besides directly perceived through morphological differentiation, the genetic diversity of different regions or the underlying mechanisms shaping local adaptation and biogeography within a system has long been recognized as critical to the maintenance of viable and resilient populations (Wiens, 2011).

Studies of the genomic heterogeneity and environmental variation of fish populations have been restricted to model species, such as salmonids (Narum et al., 2017), African cichlids (Brawand et al., 2014), and the three‐spined stickleback (*Gasterosteus aculeatus*) (Jones et al., 2012). As a nonmodel species, *Hyporhamphus intermedius* (Family Hemiramphidae), an anadromous halfbeak, is likely to be considered a novel system for this study, with a large geographic range in the estuaries of Mainland China from the East China Sea to the South China Sea (Collette & Su, 1986; Li & Zhang, 2011). The species tolerates different evolutionary efforts on an increasing geographical scale and shows tangible evidence of strong adaptive capacity (Chen et al., 2018). The populations in dissimilar habitats show broad phenotypic plasticity and environmental tolerance (Crozier & Hutchings, 2014; Rajkov et al., 2018). Preliminary investigations identified extensive morphological diversification across its range, with possible cryptic lineages within groups (Collette & Su, 1986). There is marked south–north divergence of its morphological traits. For example, the rays of the dorsal and anal fins differ and the northern population has significantly more vertebrae than the southern populations (Collette & Su, 1986). Some halfbeaks are found in the Yunnan Plateau Lakes (the upper reaches of the Pearl River and hundreds of miles inland), as a result of introductions in the 1980s (Zhu & Chen, 1989); however, the origins of the introduced populations remain unclear. The Plateau Lakes have a unique set of environmental conditions, including a wide altitudinal gradient of temperature and low dissolved oxygen (DO), which could lead to the rapid differentiation of the introduced populations (Cui et al., 2008; Fan et al., 2011; Sihai et al., 2013).

Although the signature of morphological differentiation among ecotypes has been demonstrated in *H. intermedius* (Collette & Su, 1986), the increasing integration of ecological factors, morphological and molecular approaches for assessing the diversity, evolutionary history, and phylogeography will foster a better understanding of differentiation and speciation in freshwater (Alter et al., 2016). Molecular genetic approaches can enhance our understanding of the processes underlying speciation in heterogeneous freshwater systems (Nosil et al., 2017). Double‐digest restriction‐site‐associated DNA sequencing (ddRAD‐Seq) involves the genome‐wide sequencing of reads for thousands of orthologous loci when no reference genome is available (Andrews et al., 2016; Peterson et al., 2012). It is a robust approach for disentangling the evolutionary history of species with high dispersal. For instance, the high rates of mutation experienced by the genome allow more rapid accumulation of mutations, and more polymorphic loci could result in better resolution when trying to differentiate populations and identify close relatives and local adaptation with potential dispersal events among populations (Kyriakis et al., 2019; Maroso et al., 2018). Based solely on morphological assessments, our previous estimates of *H. intermedius* diversity indicated the presence of intraspecific phenotypic differentiation.

In heterogeneous environments, local abiotic parameters may have stronger effects on genetic diversity and drive the spatial distribution of freshwater species. The genetic structure and relative strength of selection are hypothesized to influence the distribution of genomic variation on different spatial scales (Forester et al., 2018; Lagisz et al., 2010). Here, we used an integrated approach combining phenotypic, environmental, and genomic data to elucidate the drivers and processes of local divergence in *H. intermedius*. This study probed the genetic diversity, demographic history, and environmental adaptation of *H. intermedius* using a ddRAD approach at a genome‐wide scale. Specifically, we addressed the following questions: (1) What is the spatial population structure of *H. intermedius* and genetic diversity at the genomic level? (2) What is the historical demography and phylogeography of *H. intermedius* in Mainland China? (3) What are the drivers of local differentiation of *H. intermedius* associated with environmental factors?

## 2 MATERIALS AND METHODS

### 2.1 Study sites and sample collection

**FIGURE 1:**
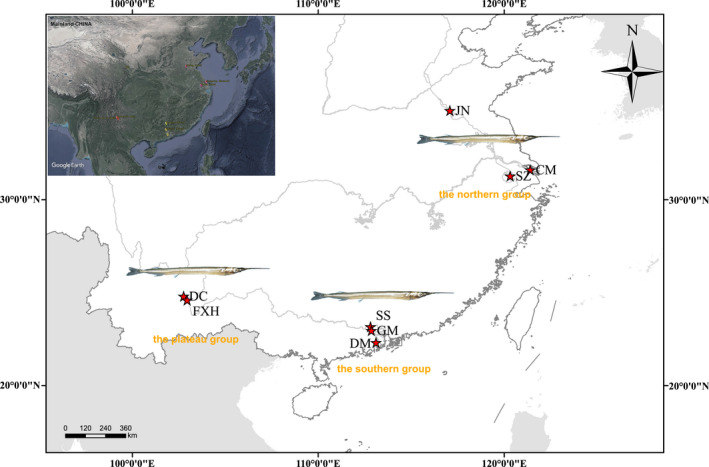
Map of the study area with locations of sampled population (SS, Sanshui; GM, Gaoming; DM: Doumen; DC, Dianchi; FXH, Fu Xianhu; CM, Chongming; SZ, Suzhou; JN, Jining); the collections are geographically divided into three groups based on environmental traits: The northern (CM, SZ, JN), the southern (CM, SZ, JN), and the plateau (DC, FXH) populations. (the map was performed by ARCGIS10.2 software, URL: https://desktop.arcgis.com/zh‐cn/)

The total samplings were collected from fish docks or local markets in 8 sites of Mainland China across three regions with different climatic conditions, from May 2017 to February 2019, including Danchi (DC), Fu Xianhu (FXH) in the plateau waters of Yunnan Province; Sanshui (SS), Gaoming (GM), Doumen (DM) in the Pearl River; Chongming (CM) near the estuary, Suzhou (SZ) in the Taihu Lake, and Jining (JN) in Weishanhu Lake (Figure 1). Populations came from heterogeneous aquatic habitats, which were defined as three geographic groups (Table S1). The map was performed with ArcGIS 10.0 software (Environmental Systems Research Institute, Inc).

**FIGURE 2:**
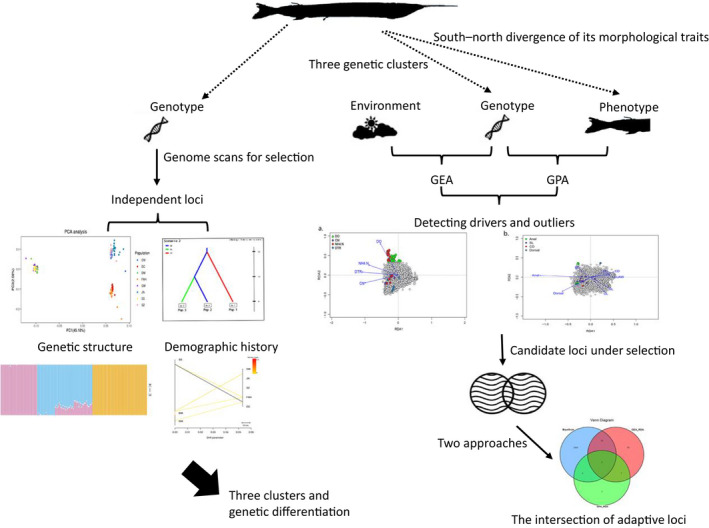
Diagram summarizing our methodological approach. We used a subset of independent loci to assess genetic structure and demographic history. Subsequently, we combined genotype, phenotype, and environmental data to identify loci under selection and then employed both Bayescan and RDA methods to determine optimal candidate loci

**FIGURE 3:**
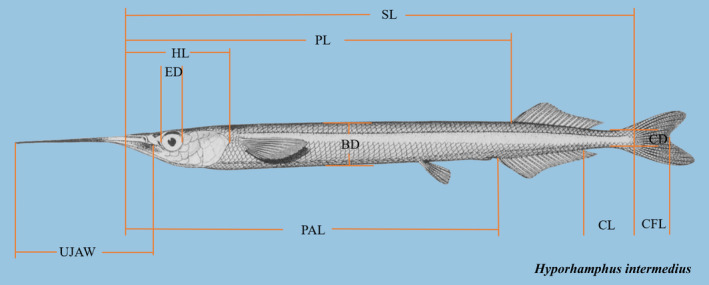
The measurement of morphological traits of *H. intermedius*. Abbreviations shown are: SL (standard length), PAL (preanal length), PL (predorsal length), UJAW (upper jaw length), ED (eye diameter), HL (head length), CL (caudal peduncle length), CD (caudal peduncle depth), BD (maximum body depth), CFL (caudal fork length). Adapted from “The halfbeaks (Pisces, Beloniformes, Hemiramphidae) of the far East” by Collette BB. And Su J. 1986; proceedings of the Academy of Natural Sciences of Philadelphia, 138, p. 259. Copyright 1986 by Academy of Natural Sciences

To elucidate the drivers and processes of local divergence in *H. intermedius*, we integrated phenotypic, environmental, and genomic data (Figure 2). A total of 156 living or dead *H. intermedius* individuals (approximately 20 per population) were selected for processing to ensure sufficient sample size, and *Hyporhamphus quoyi* (*n* = 3) were collected as outgroup. The living samples were transferred into 10 L aquaria, which was dissolved with a high concentration of 150 mg/L MS‐222 (Beijing Green Hengxing Biological Technology Co). All individuals appeared deep anesthesia or death behaviors (lost balance and sank into the bottom of aquaria) during 1–2 min, and the surgery and sampling were made after a respiratory arrest. We measured their standard length (SL), weighed them on an electronic balance (body weight: BW), and then took a lateral photograph of the left‐hand side of body shape to measure 10 morphological traits and perform geometric‐morphometric analyses with Digimizer v.4.5.1 (http://www.digimizer.com) (Figure 3). All dorsal and anal fin‐rays were counted under a dissecting microscope. Then, the fins or muscles were sampled and transferred to the laboratory in liquid nitrogen and then stored at −80 °C for further experiments.

### 2.2 RAD data processing

Genomic DNA was extracted from fin or muscles of each individual from each of 8 populations using the CTAB (Doyle & Doyle, 1987) method. Then, double‐digest restriction‐associated DNA (ddRAD) was conducted as the protocol described by Peterson et al. (2012) and libraries were prepared using 500 ng of DNA per sample. Genomic DNA was digested with the restriction enzymes *EcoR1* and *BfaI* at 37 °C for 5 h followed by the ligation of the original Illumina adapter sequences and unique 8 bp barcodes that were used for library preparation. The quality and quantity were assessed via gel‐electrophoresis on 1.5% agarose gels, where 220–450 bp long fragments were size selected, then purified using a Zymoclean Gel DNA recovery kit. Each pool was incubated at 14 PCR cycles in 25 μl reactions, which contain 5 μl 5 × Reaction buffer, 5 μl 5 × High GC enhancer, 0.25 μl of Q5 polymerase, 4 μl of library DNA and a unique indexing primer for each pool corresponding to the standard Illumina multiplexed sequencing protocol.

The ligation products were amplified in PCRs using a Veriti 96‐well thermal cycler (Life Technologies) and the protocol consisted of initial denaturation at 98°C for 30 s, 14 cycles (98°C for 15 s, 65 °C for 30 s and 72°C for 30 s), followed by a final step at 72°C for 5 min. DNA libraries were quantified using a 2100 Bioanalyzer (Agilent Technologies). Finally, pools were combined in equimolar concentration to form a single genomic library and sequenced on an Illumina HiSeq 2500 using 150 bp pair‐end reads.

### 2.3 Population genetic and statistical analyses

All raw sequences from the Illumina HiSeq lanes were checked for initial quality using FastQC, SNPs were detected using the de novo pipeline in Stacks v1.3.5 (Catchen et al., 2011, 2013). Sample reads were trimmed and orthologous loci were assembled using *ustacks* with a minimum depth of coverage required to create a stack (m) of five and four maximum. The catalog of loci was assembled using cstacks and matching rad‐tags used the *sstacks* program. Finally, *populations* program was used to filter and output the SNP sites of all samples. Loci were considered as valid if they were present in at least 75% of individuals with a minor allele frequency (MAF) > 0.05. The *population* script was applied to calculate population genetic diversity parameters such as nucleotide diversity (π), expected and observed heterozygosity (He and Ho), and F‐statistics including genetic differentiation (*F*_ST_) and average genetic differentiation between individuals within their sampling locations (F_IS_) were estimated with Stacks v1.46 (Catchen et al., 2013). We exported SNPs from the population's module in Stacks with the write_single_snp option. We restrict data analysis to only the first SNP per locus to filter for linked loci A hierarchical analysis of molecular variance (AMOVA) was used to estimate source of variation at three hierarchical subdivisions (among groups, among populations within a group, and within the populations) by Arlequin v.3.5 (Excoffier & Lischer, 2010).

Maximum likelihood estimates of population assignments for each individual were obtained using ADMIXTURE v.1.3 (Alexander & Lange, 2011) with a 10‐fold cross validation (CV), and this analysis assumes that loci are unlinked. To limit any possible stochastic effects from single analyses, we ran 100 iterations at each value of K (number of populations; K = 1–10). Each analysis used a block relaxation algorithm for point estimation and terminated once the change in the log‐likelihood of the point estimations increased by <0.0001. The optimum K was based on the average of CV‐errors across the 100 analyses per K value. We then used the program CLUMPP v.1.1 (Jakobsson & Rosenberg, 2007) to determine the robustness of the assignments of individuals to populations at each K value. In CLUMPP, we employed the Large Greedy algorithm and 1000 random permutations. Final admixture proportions for each K value and per sample assignment probabilities were based on CLUMPP analyses of all 100 replicates per K value.

The relationship between geographic coordinates and genetic structure was also identified in principal component analysis (PCA) using the R package “adegenet” (Jombart, 2008; Jiang, Qiu, et al., 2018; Jiang, Gardner, et al., 2018), which was defined as the first two components of the PCA. PhyML 3.1 was used to estimate maximum‐likelihood phylogenies by bootstrapping over loci 1000 times. The results were performed on iTOL (https://itol.embl.de/). Then, TreeMix 1.12 (Pickrell & Pritchard, 2012) simultaneously estimates a maximum likelihood (ML) species tree and the direction and weight (w) of gene flow among taxa based on allele frequencies. An ML species tree without migration is built first, and then migration events are sequentially added until the ln (Likelihood) is maximized. To test between tree models with and without gene flow we applied a likelihood ratio test.

**FIGURE 4:**
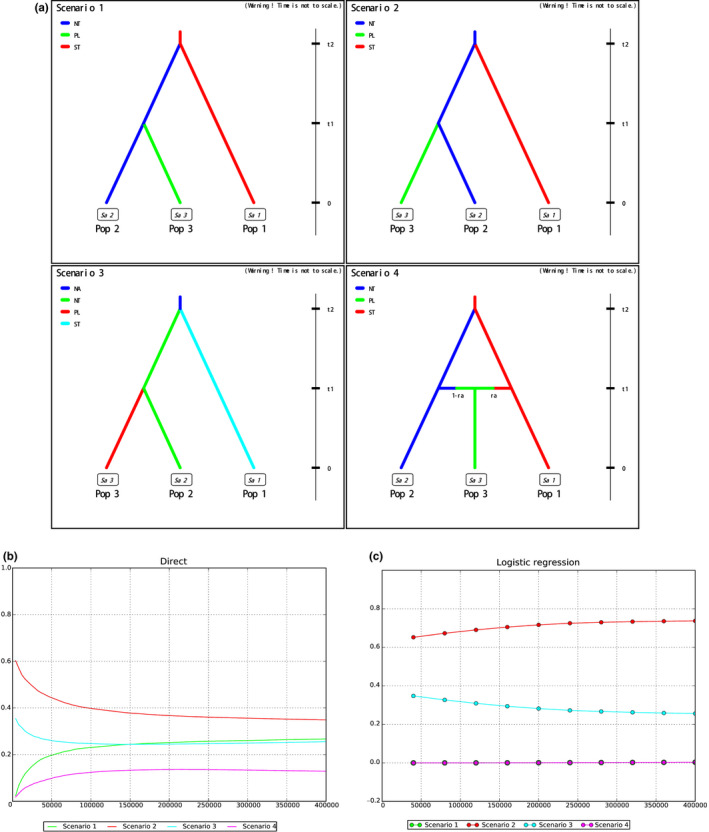
Competing scenarios were designed for inferring the demographic histories for ABC analysis. (a) Considering the results of ADMIXTURE, and phylogenetic analyses, we classified the 8 population into the following three regional groups: Pop1 represents the southern population; Pop2 the northern populations: Pop 3 the plateau populations. (b) Model comparison step using the direct approach (Miller et al., 2005). (c) Model comparison using the logistic regression approach (Beaumont et al., 2002). Three parental populations with constant effective populations sizes NT, ST, and PL. in scenario 3, two parental populations with constant effective populations sizes populations sizes NT and ST have diverged at time t2 from an ancestral population of size NA

We examine neutrality test to detect past demographic expansion, and Tajima's D values (Tajima, 1989) were calculated with sliding window (window‐size = 10,000 bp loci). Approximate Bayesian computations were executed in DIYABC v2.1 (Cornuet et al., 2008), to infer the most likely ancestral source for each geographic group. Four competing scenarios were designed where samples from the ancestral native range data were clustered into three lineages and two main ancestral sources according to our ADMIXTURE results and geographic location. Our scenarios tested whether invasive populations of the Plateau group originate solely from the Northern group (Scenarios 1, 2, 3), or are an admixture with the Southern group (Scenario 4, which includes an admixture event where the admixture rates “ar” and “1‐ar” represent the genetic contribution of each ancestral native population (Figure 4a; Table 1). Each scenario was characterized by a number of historical and demographic parameters that were expressed as the number of generations back in time. Invasive populations diverged from were northern populations “t_1_” generations ago (based on book recording), while the Northern and Southern groups were separated from each other at t2 generations ago. The ancestral native northern, southern, and plateau populations are NT, ST, and PL; NA is their common ancestral population. Priors for bottleneck duration and the effective number of founders were broad as no prior information is available.

**TABLE 1:** Prior distributions of the parameters used in DIYABC

Parameter	Probability distribution	Minimum	Maximum
Effective population size			
NA	uniform	10	100,000
NT	uniform	10	100,000
ST	uniform	10	100,000
PL	uniform	10	100,000
Time scale in generations			
t1	uniform	10	100,000
t2	uniform	10	100,000
Admixture			
ra	uniform	0.01	0.5

A total of 2 × 10^7^ simulated data sets were used with uniform prior distributions of *N*_e_ (Table 1). A stepwise‐mutation model was assumed for 469 SNPs loci with a missing rate <0.1, and summary statistics including the mean of nonzero values, and proportion of zero values for Genic diversities, *F*_ST_ distance and Nei's distances. Pre‐evaluation of each scenario was performed by PCA (Principal Component Analysis, Figure S1) within DIYABC (Cornuet et al., 2008). Both direct and logistic regression to assess posterior probabilities across 100,000 simulated pseudo‐observed data sets were chosen for comparison to enable a ranking of scenarios. To evaluate the confidence of the winner scenario, the posterior predictive error was calculated in the logistic approach. Finally, each scenario’s posterior probability was computed based on 10% simulated data closest to the observed data using a logistic regression procedure, applying logit transformation to all parameter values. The precision of each parameter estimation was evaluated by calculating the relative median absolute deviation (RMedAD) (Cornuet et al., 2010). The summary statistics were scaled by the mean effective population size of the present four populations because the mutation rates of the SNPs were unclear. Finally, we assessed a model verification step by evaluating the goodness‐of‐fit of the winner scenario concerning the observed dataset.

### 2.4 Assessing genotype‐environment and genotype–phenotype associations

We performed a redundancy analysis (RDA) to evaluate associations among the genotype, phenotype, and environment (GEA: genotype‐environment association; GPA: genotype‐ phenotype association) in driving spatial genetic differentiation (Rao, 1964). Standardized phenotypic variables (SL: standard length, Body Weight, Dorsal fin, Anal fin, PAL: preanal length, PL: predorsal length, UJAW: upper jaw length, ED: eye diameter, HL: head length, CL: caudal peduncle length, CD: caudal peduncle depth, BD: maximum body depth, CFL: caudal fork length) were identified Genotype–phenotype association, while a total of 14 environmental variables whose monthly measurements were carried out with an YSImeter (computermodule: 650 MDS, sonde: 6920; YSI Inc, Yellow Springs, OH) or collected from The China Meteorological Data Service Center (http://data.cma.cn/) was considered as potential drivers of spatially varying selection (Table S2).

RDA assumes a linear relationship between the response variables (genotypes) and the explanatory variables (i.e., environmental variables, phenotypic traits). In our case, the set of 5498 SNPs markers were converted from .vcf to. raw format using “plink” (Purcell et al., 2007). Then, the matrix was extracted using the “adegenet” (Jombart, 2008) package and all RDAs were performed using the *rda* function in the “vegan” (Oksanen et al., 2017) package in R (following Forester et al., 2018). Highly correlated predictors among environmental variables or phenotypic traits were removed by |r| > 0.75 (Dormann et al., 2013). Multicollinearity between predictors was assessed using the variance inflation factor (VIF) and since all predictor variables showed VIF <10 none were excluded. The proportion of variance in allele frequencies could be explained by environmental predictors based on the adjusted R^2^. We used an analysis of variance (ANOVA) with 1000 permutations to evaluate the significance of the global RDA. Finally, we used scaling = 3 (also known as “symmetrical scaling”) on the first two constrained ordination axes for the ordination plots (Borcard et al., 2011) and identified the environmental variables or phenotypic traits.

### 2.5 Detection of outlier single nucleotide polymorphisms

Two outlier detection methods were employed: BayeScan 2.1(Foll & Gaggiotti, 2008) was applied for the detection of loci under natural selection, which identified “outlier” loci by splitting *F*_ST_ into population‐specific components shared by all loci and locus‐specific components shared by all populations. Bayesian approach was used to find outlier (FDR = 0.01) in the dataset containing 5498 SNPs. All analyses were conducted with 20 pilot runs for 5000 iterations, followed by 100,000 iterations with a burn‐in of 50,000 steps. The default value of prior odds (10) was used. Loci with alpha‐values significantly >0 were considered as loci under directional selection while those with alpha <0 were considered as loci under balancing selection (Moore et al., 2014). All other loci were considered neutral. The output file of *F*_ST_ was loaded into R v.4.0.5 using the BayeScan package following Geweke's for convergence diagnostics and outliers' detection. We acquired the list and the number of outliers, and the plotting.

As BayeScan is sensitive to a hierarchical structure in the data causing high false positive rate, we also performed an RDA as multilocus GEA (genotype‐environment association) and GPA (genotype–phenotype association) methods to detect loci putatively under selection based on correlations with environmental variables and morphological traits. This approach can detect multilocus signatures of selection based on locus scores from significant axes (i.e., the loading of each locus in ordination space) that were ± 3 SD from the mean loading on the first two constrained ordination axes. It may have very limited power in identifying spatially diversifying selection and we also could not align candidate loci against no genomic resources for nonmodel species. In addition, the SNP loadings were stored as species in the RDA object and extracted from the three significant constrained as described above. The program parameters followed Forester's setting (Forester et al., 2018), at which candidate outliers were obtained. Outlier loci identified by two methods were considered “candidate outliers,” which were presented in Venn plotting by “VennDiagram” package within R v.4.0.5.

## 3 RESULTS

### 3.1 Single nucleotide polymorphisms from double‐digest restriction‐site‐associated DNA sequencing

We established a ddRAD library and sequenced 150‐bp paired‐ends from 156 individual fish in eight populations. This produced an average of 16,016,724 (range 12,794,576–24,139,524) raw sequence reads per sample. After removing poor‐quality sequences, an average of 15,046,821 (range 11,394,714–22,827,782) raw reads were available for sequence mapping and finding SNPs. Of the total 32,744 SNPs detected from 13,684,154 tags, 27,246 were discarded due to excessive missing data in >80% fish in collection, retaining 5498 high‐quality SNPs with minor allele frequency (MAF) > 0.05 across the entire sequence dataset.

### 3.2 Genetic diversity and population divergence

The 61,898,184‐bp genomic region amplified from 156 individuals identified 5498 SNP sites. There were slight differences in genetic diversity among groups in different watersheds (Table 2). The mean expected heterozygosity (He) of the loci over collections range from 0.1786 to 0.1973, the Northern (SZ, CM, except JN) and the Plateau (DC, FXH) had higher values than the southern populations. Similar trending was observed on nucleotide diversity, though the Plateau group were constructed after transplantation, and the positive values of F_IS_ were lowest within the plateau populations (0.0254–0.0265). We have test percentage of loci in Hardy–Weinberg disequilibrium per population (*p*‐value < .05) (Table 2), and loci in Hardy–Weinberg disequilibrium are few (<4%) in per population, while most of them (> 70%) for whole populations are in disequilibrium.

**TABLE 2:** The results of population genetic diversity index

Pop ID	Num Indv	Ho	He	π	D	F_IS_	HWD (*p* < .05)
DC	18.5578	0.1795	0.1841	0.1892	0.9523	0.0265	2.64%
FXH	17.1096	0.1811	0.1851	0.1907	0.9796	0.0254	3.73%
JN	16.9272	0.1643	0.1801	0.1856	0.7373	0.0579	1.07%
SZ	19.3163	0.1865	0.1973	0.2025	0.8397	0.0462	3.36%
CM	18.9608	0.1834	0.1928	0.1981	0.8498	0.042	2.55%
SS	18.85	0.17	0.1813	0.1863	0.9065	0.0473	1.87%
GM	17.6987	0.1697	0.1807	0.186	0.9105	0.0448	3.33%
DM	17.7985	0.1661	0.1786	0.1838	0.8198	0.0513	2.04%

^§^ The population genetic statistics includes Num Indv: average number of nucleotide differences; π: nucleotide diversity; He: expected heterozygosity; Ho: observed heterozygosity, F_IS_: population differentiation between individuals among sampled locations. D: Tajima's D mean values by neutrality test; HWD: percentage of loci in Hardy–Weinberg disequilibrium per population (*p*‐value < .05).

An AMOVA (analysis of molecular variance) of the 5498 SNPs showed significant genetic variation among groups (56.70%) and within populations (43.01%) (Table 3). Furthermore, the pairwise *F*_ST_ values of the Southern group from the Pearl River System had a high level of differentiation (0.3171–0.3781) compared to the remaining groups (Table 4), forming a distinct cluster in the molecular phylogenetic tree (Figure S2). However, the genetic differentiation between each pair of populations within geographic groups was lower. Even though the genetic divergence of both the Northern and Plateau did not reach significance (0.0372–0.0553) in the last decades, the pairwise *F*_ST_ were slightly increased, indicating the tendency toward genetic differentiation.

**TABLE 3:** AMOVA analysis of 8 populations based on 5498 SNPs sequences

Source of variation	d.f.	Sum of squares	Variance components	Percentage of variation	*p*‐value
Among groups	2	12078.3790	44.4953	0.5670	.0001
Among populations within groups	5	5027	0.2255	0.0029	.3351
Within populations	149	5231.0000	34	0.4301	.0001
Total	156	22564.8260	78.4692		
*F*_ST_	0.5699				

**TABLE 4:** Pairwise F_ST_ among 8 populations based on 5498 SNPs sequences

Population	DC	FXH	JN	SZ	CM	SS	GM	DM
DC	0							
FXH	0.0218	0						
JN	0.0510*	0.0553*	0					
SZ	0.0375	0.0409	0.0237	0				
CM	0.0372	0.0401	0.0277	0.0155	0			
SS	0.3373*	0.3349*	0.3781*	0.3171*	0.3208*	0		
GM	0.3382*	0.3359*	0.3760*	0.3183*	0.3224*	0.0157	0	
DM	0.3469*	0.3391*	0.3697*	0.3231*	0.3271*	0.0155	0.0153	0

* *p* < .05.

### 3.3 Genetic structure, phylogeography, and migration

In the phylogenetic analysis, the Southern group (SS, GM, and DM) formed a distinct clade that was separated from the other populations with 100% bootstrap. The individuals from the Northern and Plateau Lakes systems were assigned to close clades, suggesting a close relationship between the two systems (Figure S2). Notably, 8 individuals from 5 populations were grouped as sister clade, providing strong evidence for transplant event.

**FIGURE 5:**
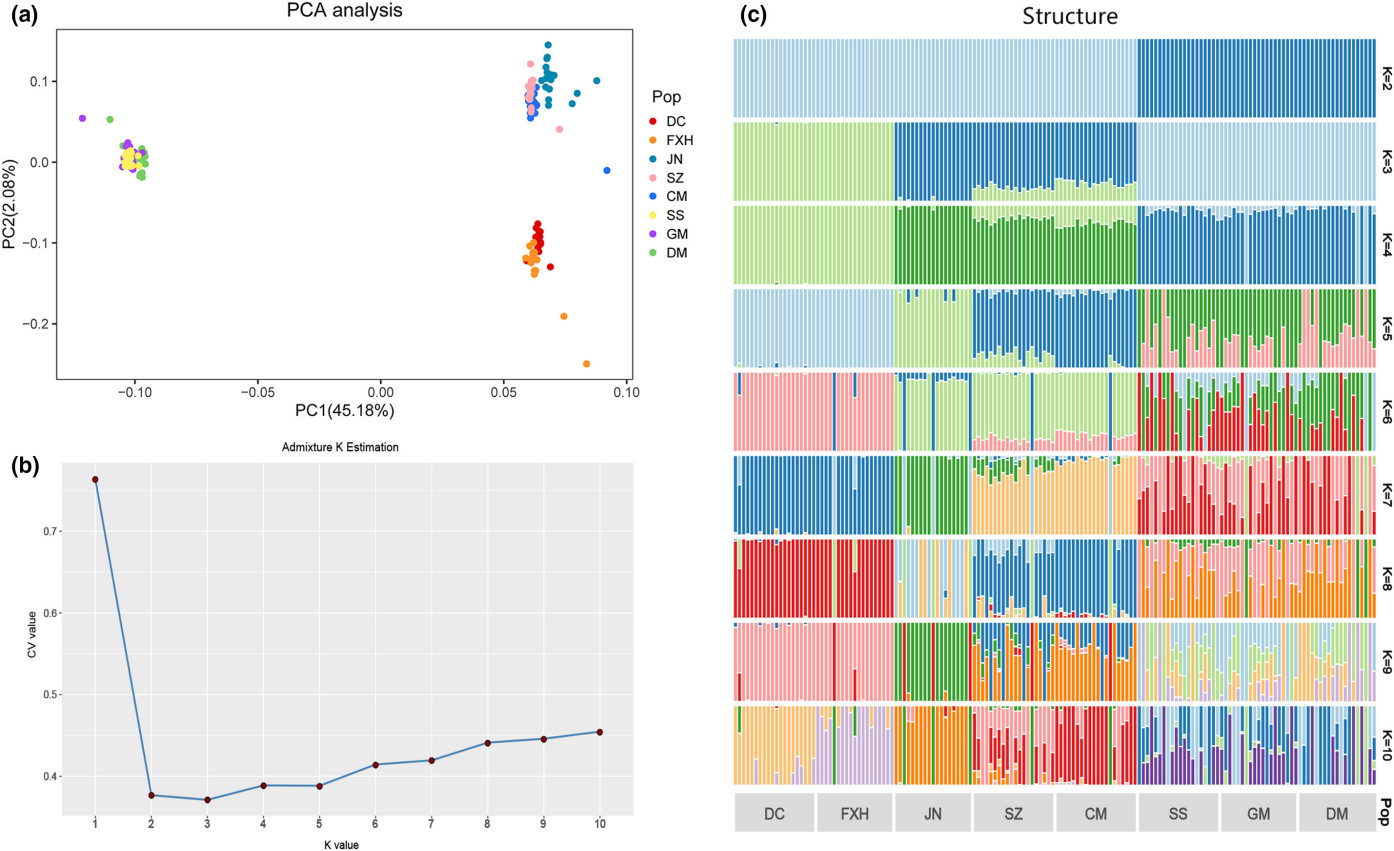
Genetic structure analysis of *H. intermedius* was revealed by principal components (PC) analysis (top panels) and ADMIXTURE (bottom panel). (a) First two PC axes. Points are colored by 8 populations. (b) Analysis of appropriate K value (c) admixture proportions of all clusters identified with ADMIXTURE. Vertical bars represent individuals and the heights of different colors point to probabilities assignment each cluster

Principal components analysis and ADMIXTURE analyses produced a similar phylogenetic topology. The first two principal component axes clearly separate three distinct lineages from each other in PCA plot (Figure 5a), which was broadly congruent at population structure. ADMIXTURE analysis suggested three genetic clusters (K = 3) (Figure 5b, c). The barplot at secondary optimal value of K = 2 provided an additional, interpretable resolution of population structure, in which the northern and plateau populations are assigned to one group, reflecting the close relationship among them.

**FIGURE 6:**
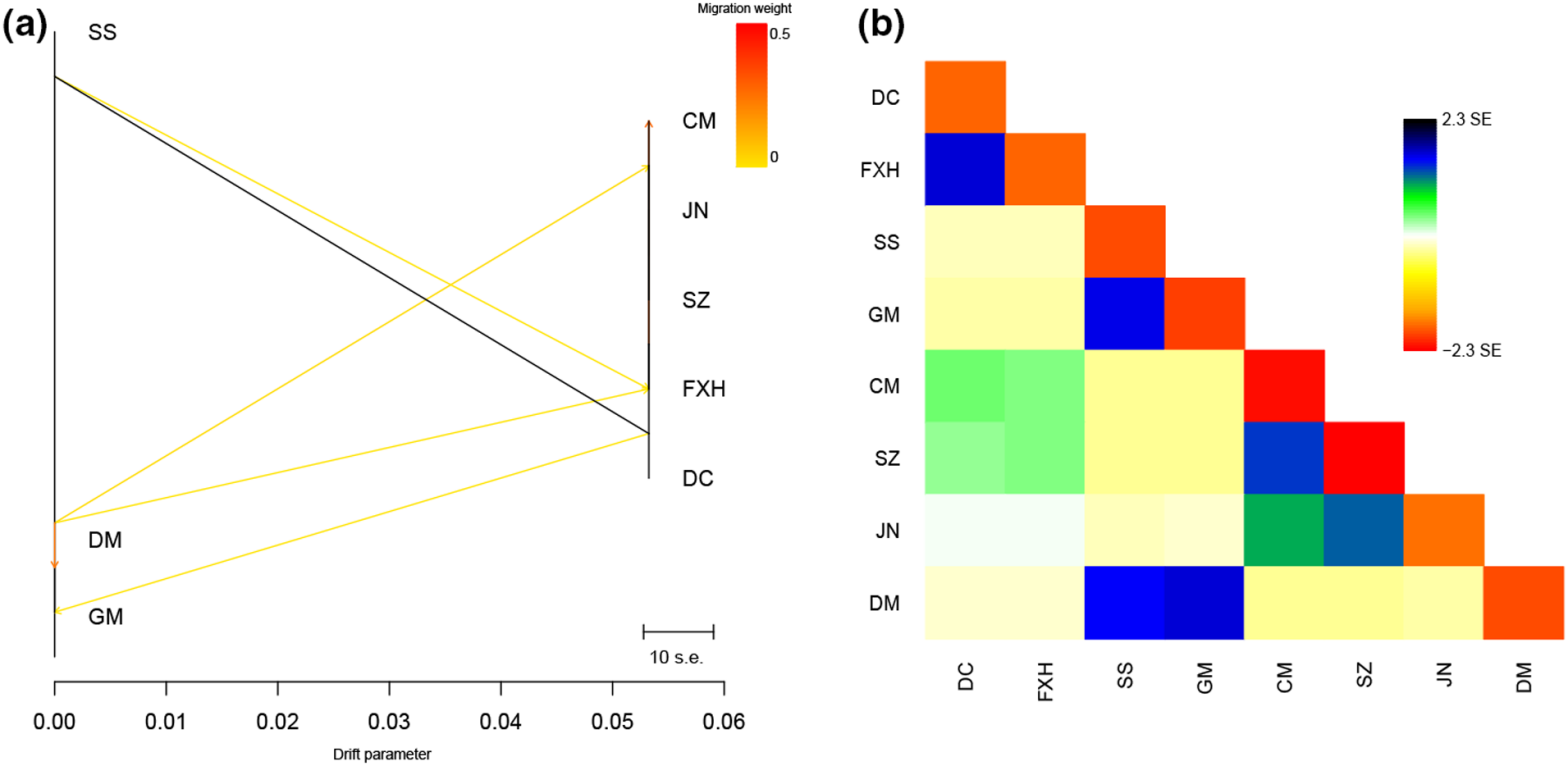
Admixture graph constructed with TreeMix using SNP data. (a) Maximum likelihood tree. Branch lengths are proportional to the evolutionary change (the drift parameter) and terminal nodes were labeled with population codes (see Table 2). The scale bar represents 10 times the average standard error (s.e.) of the values in the covariance matrix, and the migration weight represents the fraction of ancestry derived from the migration edge. (b) The residual covariance between each pair of populations from the maximum likelihood tree. Colors are described in the palette on the right. Residuals above zero represent populations that are more closely related to each other in the data than in the best‐fit tree, and thus are candidates for admixture events

TreeMix analyses were used to estimate the direction and magnitude of gene flow among taxa. The unrooted phylogenies revealed two key results: first, within the two main clades, the Southern group were well separated from the remaining sites, while the Plateau and northern groups were admixtures with similar groupings of taxa to those found in the secondary optimal structure (Figure 6a). Next, a number of populations were identified as likely candidates for recent admixture events (Figure 6b). Gene flow was inferred for several pairs of taxa, including gene flow, for instances, from the FXH into the CM, or migrations between pairs of Southern sites. Six historical migration events were added to the tree sequentially, and two of them were detected as high weight.

### 3.4 Demographic history of the ancestral populations

To assess the deviation from neutrality across groups, the results using the complete SNP dataset of *H. intermedius* had a significant positive value (mean Tajima's D = 1.936; Figure S3), which revealed that medium‐frequency loci were polymorphic and dominated the genomes, indicating the effects of population subdivision, which maintained the higher genetic variance.

Four possible scenarios of ancestral origin of *H. intermedius* were implemented in DIYABC (Figure 4a). The analysis showed that scenario 2 (Both the southern populations and the Plateau populations deriving from ancestral native northern populations) had the highest support, with a posterior probability of 0.348 with the direct approach (Figure 4b) and 0.737 with the logistic approach (Figure 4c), while the other models had lower probabilities. Most parameter estimates showed high RMedAD values (>0.2) and cannot be considered fully reliable (Table S4). Based on the demographic model, the scaled model values of effective population size varied greatly among regions: the northern populations (NP) were large (8.69E+03), while the plateau (PL) was smaller (6.42E+03), respectively, and the southern population (SP) was only 7.17E+02 (Table S3).

### 3.5 Genotype‐environment and genotype–phenotype associations

**FIGURE 7:**
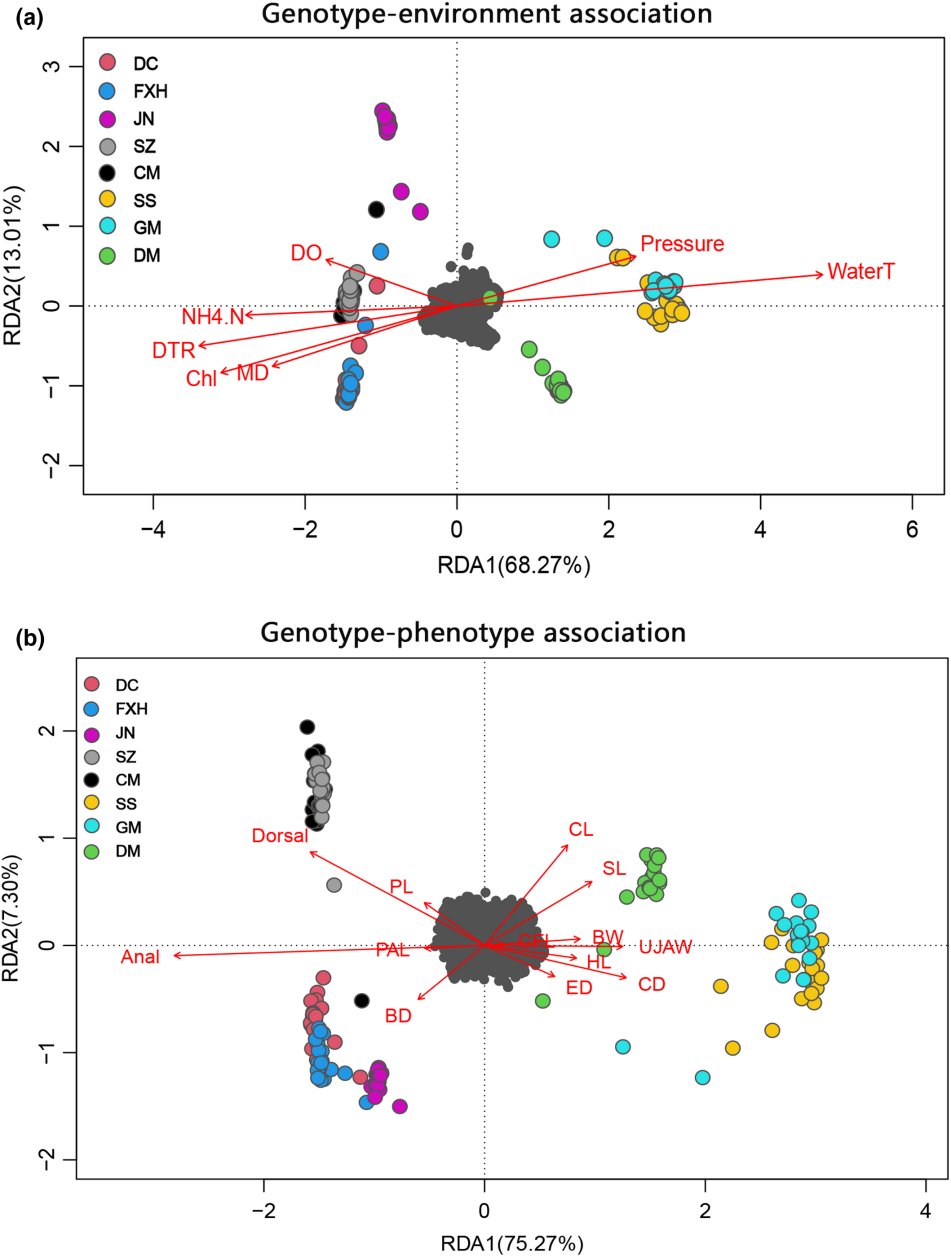
Redundancy analysis (RDA) performed with 5498 SNPs and explanatory variables (red arrows) on the first two constrained ordination are shown based on locus scores±3 SD with SNPs represented as gray dots. The RDA for (a) genotype‐environment association (upper panels) and (b) genotype–phenotype association analyses (lower panels). For site codes, refer to Figure 1; for morphological trait codes, refer to Figure 3

Based on all 5498 SNPs including all 8 sampling locations, redundancy analysis (RDA) of climatic and environmental variables showed that the first two components explained, respectively, 82.1%, 8.96% of the variation (Figure 7a), and four variables (dissolved oxygen, chlorophyl a, NH4 + ‐N, and daily temperature range) were selected and showed statistically significant variation with genotypes (*p* = .001, *R* ^*2*^_*adj*_ = 0.2938). As predictive of genetic variation, all variables excluding dissolved oxygen (DO) were completely negative correlation to axis 1 in the RDA, whereas DO have the largest squared constraint score in the second RDA axis (Table 5), suggesting this variable contributes the most to SNP variation. Interestingly, the first and third axes split individuals into three large genotype‐environment association (GEA) groups corresponding to their sampling location.

**TABLE 5:** Redundancy analysis summary statistics of genotype‐environment association

	RDA1	RDA2	RDA3	RDA4
Eigenvalues	1408.36	153.72	122.15	31.19
Proportion Explained	0.821	0.0896	0.0712	0.0181
Cumulative Proportion	0.821	0.9106	0.9818	1

Environmental variables	*p*‐value	*r* ^2^	Vif.cca
DO	.001	0.7052	1.354
Chlorophyl a	.001	0.4039	1.234
NH4 + ‐N	.001	0.3242	1.098
Daily temperature range	.001	0.4144	1.477
*R* ^*2*^_*adj*_	.2938		

Pearson analysis distinguished pronounced changes in shape associated with the bases of the dorsal and anal fins (Figure S4), indicating that the appearance of a dramatic phenotypic difference coincided with the genomic differentiation. In *H. intermedius* genotype–phenotype association (GPA) models, ten phenotypic traits identified using RDA exhibited statistical association (*p* = .001, *R* ^*2*^_*adj*_ = 0.2438; Table S5), when constrained by morphology. The first RDA1 axes represented the main variance (76.74%) in SNP genotypes, followed by precipitation in RDA2 (7.42%) and RDA3 (4.95%) (Figure 7b, Table S5). Anal and dorsal fins were core variables to uncover separation between Southern group and remaining populations (*R* ^*2*^_*anal*_ = 0.7269, *R* ^*2*^_*dorsal*_ = 0.4046), supporting a role for anal and dorsal differences facilitating divergence between ecotypes.

### 3.6 Outlier loci under selection

**FIGURE 8:**
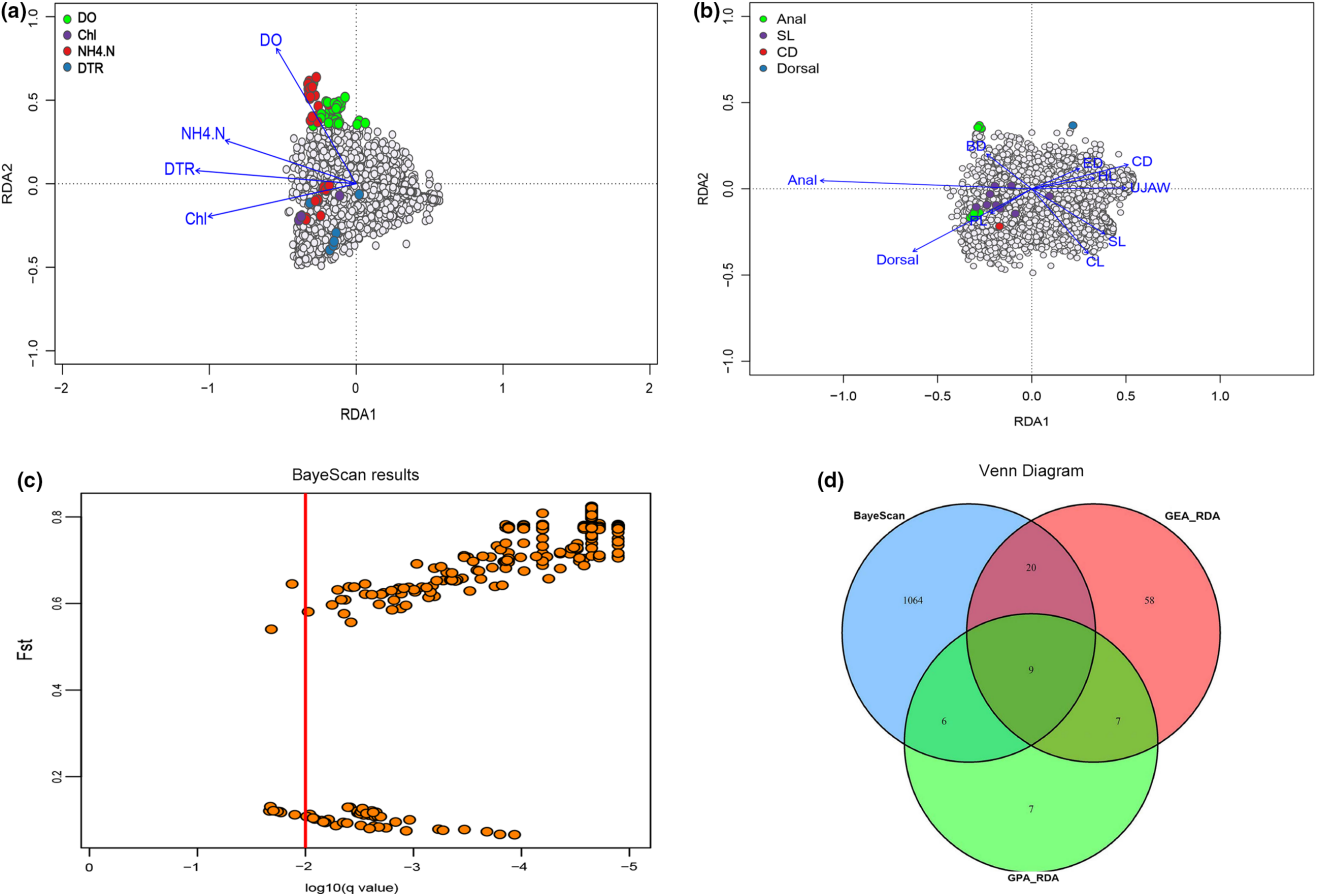
Results from detecting the candidate outliers. (a, b) Candidate outliers of GEA and GPA using redundant analysis (RDA) are shown as colored points based on locus scores ±3 SD from the mean loading on each RDA axis. The plots show the first three constrained axes from RDA, with SNPs represented as gray filled circles, environment and phenotype variables as blue arrows, and individuals from different population in color circles. (c) Results from BayeScan analysis illustrating neutral and selective SNPs. The vertical line represents the corresponding to an FDR = 0.01, to the right of which potentially loci under selection are shown. (d) Venn diagram showing the intersection of candidate SNPs detected by BayeScan and RDA (GEA_RDA & GPA_RDA) approaches

Using the Bayescan approach, 1099 loci were retained as outliers to be under selection. One thousand and fifty‐five of these SNPs had significantly higher *F*_ST_ than the background of the genome (i.e., they are putatively under diversifying selection) and 49 have significantly lower *F*_ST_ than the background of the genome (i.e., they are putatively under balancing/purifying selection; Figure 8c). Using RDA models, we identified a total of 94 showing GEA and 29 loci in GPA (Figure 8a). In GEA, the majority of loci were most correlated to NH4 + ‐N (47) and DO (34). Of the remaining outliers, seven were associated with mean DTR and six to the content of chlorophyl a; while in GPA, a large proportion of SNPs was more specifically attributed to anal fins and standard length in morphology, respectively (Figure 8b). Despite no direct way of aligning detected outliers, the Venn diagram revealed only 16 outliers contained both GEA and GPA. Combining both Bayescan and RDA methods, a total of nine of the SNPs were detected as being under diversifying selection, which was more likely to be candidate loci (Figure 8d).

## 4 DISCUSSION

### 4.1 Population structure and phylogeographic relationships

Unlike marine environments, freshwater fish are typically influenced by various geographical factors and often have lower gene flow between hydrographic basins (Bloom et al., 2013; Puebla, 2009). There is no information on the interaction among regional populations of *H. intermedius*, as anadromous *H. intermedius* is stopped by geographic constraints, without multi region gene flow. Genetic differentiation can arise as a result of geographic barriers, which can potentially give rise to genetic structure (Fan et al., 2019; Wright, 1950). In our study, the ddRAD‐Seq analyses showed evident spatial genetic structure and reflected its geographic arrangement in Mainland China. Genetic differences are expected to occur when fish in different habitats require unique abilities to survive (Bourke et al., 1997). We identified genetic‐separated groups of *H. intermedius* on the mainland though it is different in the PCA and phylogenetic analyses. Our genetic results agreed with the classic geographic scenario in which these lineages are significantly differentiated genetically among different regions and the intraspecific lineage of species is often reported to be differentiated in different environments, such as climate change arising by latitude and altitude (Domrös & Peng, 1988; Jiang, Qiu, et al., 2018).

Of note, there are only two clusters on PC1 and only 2% of the variation on PC2 in PCA analyses, though the optimal value of genetic structure was K = 3. The best explanation was Plateau group was closely related to the Northern group, as shown by the analysis of gene flow. Importantly, these different findings provide evidence to its phylogeographic relationships (Zhang et al., 2012). The cause of the high degree of relatedness between the groups is likely due to the short time since transplantation. Considering the geographic barriers among the river systems and that each group is separated by >1000 km, we observe that the pairwise population genetic differentiation was complicated, as might be expected. Strong within‐group migration still existed, although some populations are widely separated, such as CM and SZ, SS and DM. These findings imply that *H. intermedius* has much greater gene flow than previously thought. Overall, the complex phylogeographic relationship suggests that *H. intermedius* diversified throughout the system and the effects of anthropogenic intervention and geographic constraints led to the current biogeographic distribution and population structure.

### 4.2 Genetic differentiation and cryptic lineages

The level of genetic diversity reflects a population's viability, meaning that a population with a higher level of genetic diversity has greater adaptability, which is conducive to its long‐term survival (Frankham et al., 2002; Reed & Frankham, 2003). The first goal of this study was to identify genomic variation of *H. intermedius* and what is about the genetic diversity. The levels of genetic diversity we observed were most likely related to spatial variation. At the genomic level, the degree of polymorphism within populations varied from 0.1838 to 0.2025; the southern populations had comparatively lower levels than northern ones. Strong geographic clustering of populations was observed, corresponding to our AMOVA estimation (among‐group variance of 56.70%). Furthermore, the pairwise *F*_ST_ analysis revealed high differentiation in the Southern group, possibly cryptic subspecies (*F*_ST_ >0.25, Wright, 1990), on the mainland, while subtle genetic differentiation (very low and nonsignificant *F*_ST_ values) between the native and introduced populations (the North and the Plateau). Strong divergence in *H. intermedius* appeared along the north–south axis based on genetic analysis, whereas Collette and Su (1986) resolved these phylogeographic relationships using morphological data with many fixed differences. Such differentiation could potentially be caused by evolutionary forces (selection or genetic drift) and was reported in the sister taxon. Analysis of deviations from the HWE on whole genomic datasets has shown that loci in all populations were more frequently deviating (> 70%), while loci in Hardy–Weinberg disequilibrium are few (<4%) per local population, which may be indicative of strong evolutionary force on whole populations. Given the presence of geographic constraints to population dispersion, we hypothesized that these lineages represent incipient species. The effects of demographic history on genetic variation highlighted the genome variation between north and south has alternatively been interpreted northern origin of *H. intermedius*, in agreement with the ABC results, as addressed in several other animal investigations (Nuñez et al., 2011).

### 4.3 Origins of the early introduced populations

The invasive populations in plateau lakes were reported to have a lower allelic diversity compared to indigenous populations, because the latter may be richer in rare alleles. Our observations are in disagreement with these researches in invasive populations developed from transplantation. The plateau populations are developed through just four decades reaching a normal level in genetic backgrounds, which was unexpected, evidencing that these clusters did not experience strong bottleneck effects after diverging from ancestral populations and have been maintained as relatively large and stable populations gathered different lineages from the north, which have direct management implications. The phylogenetic pattern observed from numerous SNPs at a genome‐wide scale manifesting the hydrology of the Yunnan–Guizhou Plateau appears to have played a central role in the genetic differentiation and structural diversification of the Plateau group following the initial colonization. Specifically, individuals from the Northern group form a lineage that resolved as native populations genetically proximate to the introduced group, as evident in the phylogeographic analysis and demographic inference and demonstrated by the history record (Zhu & Chen, 1989). Clear evidence of admixture as reflected in the Treemix result is present in the whole genomes, although the northern populations are more distant from the Plateau Lakes and isolated by various continental barriers. Historically, *H. intermedius* was first introduced into the Plateau Lakes in Yunnan Province in the 1980s, although there is no clear record of the route involved (Xiong et al., 2006). The introduction routes of the plateau populations can be inferred from several investigations; most may have originated from Jiangsu Province (Xiong et al., 2008; Zhu & Chen, 1989), which overlaps the Yangtze River Basin.

The genetic differentiation among the native populations also provided clues to the origins of the introduced plateau populations (Cornuet, 1999; Shen et al., 2019; Waples & Gaggiotti, 2006). Both genetic differentiation and an obvious geographic pattern to the population distribution in the diverse aquatic environments were identified in a background of migration. The introduced populations in the Plateau Lakes formed an independent genetic cluster, with a few individuals admixed with the Northern group. Of note, the slight genetic divergence of the Plateau and the Northern was unexpected. The Plateau Lakes are geographically adjacent to the Pearl River System, so that the Plateau and the Southern were hypothesized to have high probability of introgressive hybridization events. However, this scenario had the lowest support among the four competing scenarios in the ABC analysis. In both the pairwise *F*_ST_ and phylogenetic analyses, the introduced populations shaped the patterns across the recent divergence. Similar conclusions are drawn from other fish research, such as in salmonids (Narum et al., 2017) and grass carp (Shen et al., 2019).

### 4.4 Local diversification in heterogeneous environments

The combination of morphological characters and genomic data, together with the environmental heterogeneity elucidated a scenario of diversification in *H. intermedius*. We identified several GEA related to environmental variables, which is consistent with results in other fish species, such as salmonids (Bekkevold et al., 2019; Bourret et al., 2013; Chen et al., 2018). Interestingly, the study shows the Plateau sampling sites that were located at the same latitude but different altitudes with the south showed strong relationships with the DO content and DTR, which reflect the effects of the Plateau on temperature fluctuation and oxygen deficit (Fan et al., 2011; Mackinnon & Carey, 1996), while the distribution of the Southern group was associated with the water temperature as a result of subtropical weather in the Pearl River Basin (Zhou et al., 2018). Compared to the south adapted to long summers and warm‐winters, the north group thrives in fluctuating, cold‐temperate environments. Such general relationships are not surprising given that in fishes, temperature is linked to key physiological, developmental, and behavioral processes, rendering fish highly sensitive to climatic and thermal conditions (Chen et al., 2018; Crozier & Hutchings, 2014). Stable and high temperature affects a broad spectrum of cellular components, accelerating enzymatic activity and metabolism in organism (Beemelmanns et al., 2021; Jesus et al., 2016; Jeffries et al., 2014). In GPA, we also found a difference in morphological traits (dorsal, anal fins rays, etc.), especially between the north and the south. The differentiation whether genetic or phenotypic among watersheds detected here appears to have been driven by selective pressure related to spatial environmental heterogeneity, which are important determinants of phylogeographic structure in many other species (Guo et al., 2015; Selleslagh et al., 2009), although some salmonid studies indicate stronger effect of phenotypic plasticity rather than adaptation to specific habitat (Solberg et al., 2016).

In fact, GEA studies can only be indicative of drivers underlying local adaptation, confirmation of functional, and adaptive significance of post‐identified genes requires rigorous testing, as the relatedness may be derived from an interaction or unrecognized factor that is strongly associated with one of the parameters examined (Guo et al., 2016; Lascoux & Merilä, 2013). Numbers of GEA identified with BayeScan were high but sensitive to a hierarchical structure in the data causing a high false‐positive rate, which suggests that absolute numbers of GEA identified may have been upwardly biased. By using the combined outliers detected in GEA and GPA by RDA, we narrowed down the possibilities and identified several candidate adaptive SNPs highly correlated with variables, although we could not align candidate loci against no genomic resources for nonmodel species. Irrespective of a potential bias, the identification GEA across broad expanses of genome with both methods is suggestive of locally adapted variation being pervasive throughout multiple genomic regions.

Additionally, this study showed that population subdivision stemming from environmental heterogeneity can introduce fine‐scale population structure and divergence (Mathias et al., 2001; Michel et al., 2010; Via, 2012). Identifying differentiation during population introduction and adaptation in heterogeneous environments genetically is pivotal to unveiling population structure and the mechanisms of adaption (Gamboa & Watanabe, 2019). The positive Tajima's D revealed that medium‐frequency loci were polymorphic and dominated the genomes, indicating strong effects of balancing selection or population subdivision (Simonsen et al., 1995; Tajima, 1989). As a result, species maintain more genetic variance and have the capacity to survive and adapt in diverse environments. Tajima's D in most genomic windows was positive and the effect of balancing selection appears in only local genomic regions (Figure S3). Instead, we speculate that genetic drift affected the entire population; as a result, higher genetic diversity was maintained at specific loci. There were few negative Tajima's D values or low‐frequency loci, indicating the absence of strongly positive selection. Local divergence and evolution will emerge when the strength of divergent selection overrides random genetic drift and the homogenizing effect of gene flow among populations (Ahrens et al., 2018; Leinonen et al., 2013). Nevertheless, there have been few direct observations of selective regions across genomes in nonmodel species. Population subdivision might promote the survival of isolated populations in heterogeneous environments and potentially affect the boundaries of local populations, which determine their phenotypic polymorphism, and wide adaptability is a good material for study.

## 5 CONCLUSIONS

Our results indicate that hydrological barriers and abiotic factors (DTR, DO, NH4 + ‐N, and chlorophyl a) mold phylogeography and diversification at large scales. Genome‐wide SNP data gave insights into the complex spatial genetic structure (three clusters) and the demographic history of *H. intermedius* in Mainland China. This uncovered the genetic differentiation in three heterogeneous hydrobiological regions, which served as a source of genetic polymorphisms for divergence and adaptation. Furthermore, we showed that the introduced halfbeak populations in Plateau Lakes originated from the Northern, whereas significantly early divergence occurred in both genotype and phenotype in the Southern group, which is an isolated lineage that may develop into incipient species. Isolation by distance was detected among southern–northern groups, and we demonstrated that *H. intermedius* underwent subdivision, which is also likely indispensable to survival during adaptation to new habitats. We offer robust evidence of disappearing migratory behaviors, the formation of population structure in *H. intermedius*, and divergence in a heterogeneous environment due to the interaction of population subdivision, as well as strong effects of abiotic factors.

## AUTHOR CONTRIBUTIONS

**Gongpei Wang:** Conceptualization (lead); data curation (lead); formal analysis (lead); investigation (lead); methodology (lead); software (lead); validation (lead); visualization (lead); writing – original draft (lead); writing – review and editing (equal). **Han Lai:** Data curation (equal); investigation (equal); validation (equal); visualization (equal). **Sehng Bi:** Investigation (equal); methodology (equal); resources (equal). **Ding‐Li guo:** Conceptualization (equal); investigation (equal); software (equal). **Xiaopin Zhao:** Methodology (equal); validation (equal); writing – original draft (equal). **Xiaoli Chen:** Data curation (equal); resources (equal); validation (equal). **Shuang Liu:** Investigation (equal); methodology (equal); writing – original draft (equal). **Xuange Liu:** Methodology (equal); validation (equal). **Yuqin Su:** Methodology (equal); validation (equal). **Huadong Yi:** Methodology (equal); validation (equal). **Guifeng Li:** Conceptualization (equal); funding acquisition (equal); investigation (equal); project administration (equal); resources (equal); supervision (equal); validation (equal); writing – review and editing (equal).

## CONFLICT OF INTEREST

The authors declare that they have no competing interests.

## Supporting information


**Figure S1** The PCA results of pre‐evaluation of each scenario. Each (small) dot represents a simulated dataset from the reference table and the large yellow dot represents the observed data set. The initial components of datasets are the values of the summary statistics from which the principal components are computed.

**Figure S2** Phylogenetic networks of *H. intermedius* represented with the maximum‐likelihood method. The NJ tree was constructed using SNPs dataset. The bootstrap values (>0.7) are given above/below the branches.

**Figure S3** SNPs in the unit sliding window corresponds to Tajima’s D value. Blue line represents the average of Tajima’s D value of SNPs, Tajima’s D = 1.936; Red line represents a dividing line, Tajima’s D = 0 observed variation equal to expected variation.

**Figure S4** Pearson correlation of 13 standardized phenotypic variables.

**Table S1**Names of sites sampled, abbreviations of their names, location, and numbers of *Hyporhamphus intermedius* used in the genomic analyses

## Data Availability

All data are incorporated into the article and its online supplementary material.
