# Supplementary figures and images for: ddRAD‐Seq reveals evolutionary insights into population differentiation and the cryptic phylogeography of Hyporhamphus intermedius in Mainland China
Source: Ecol Evol. 2022 Jul 4;12(7):e9053. doi: 10.1002/ece3.9053 (PMC9251877; doi:10.1002/ece3.9053)

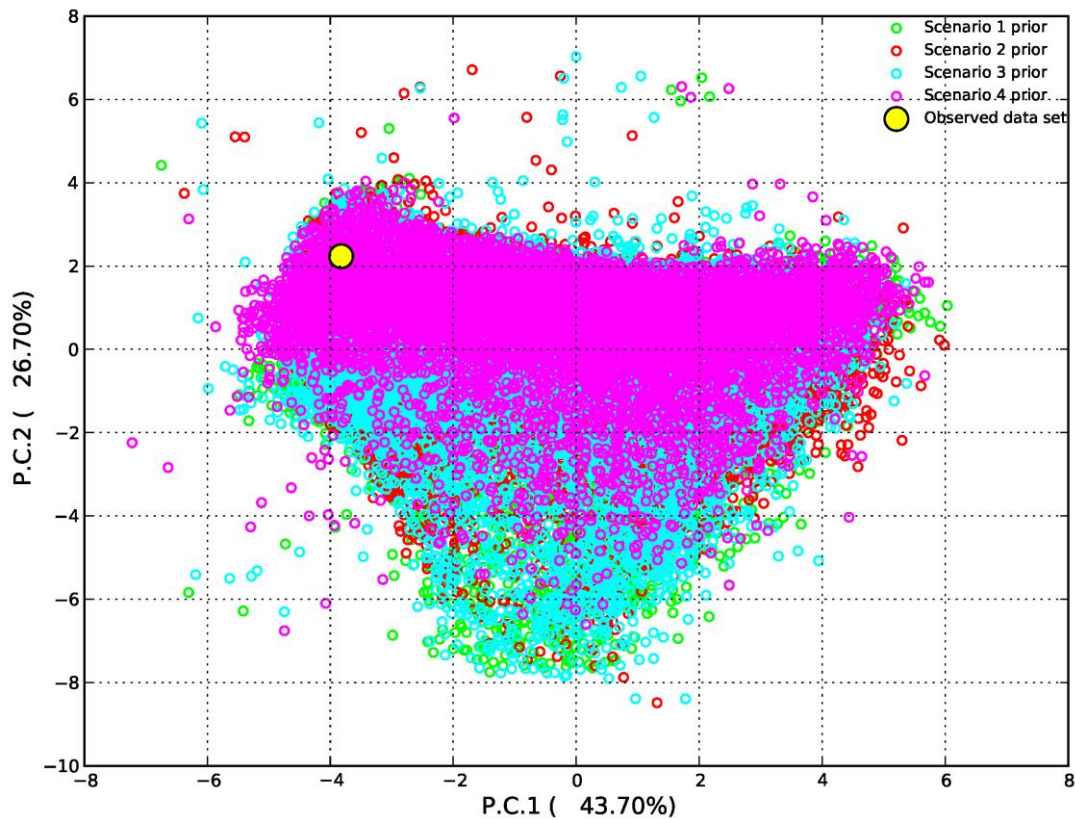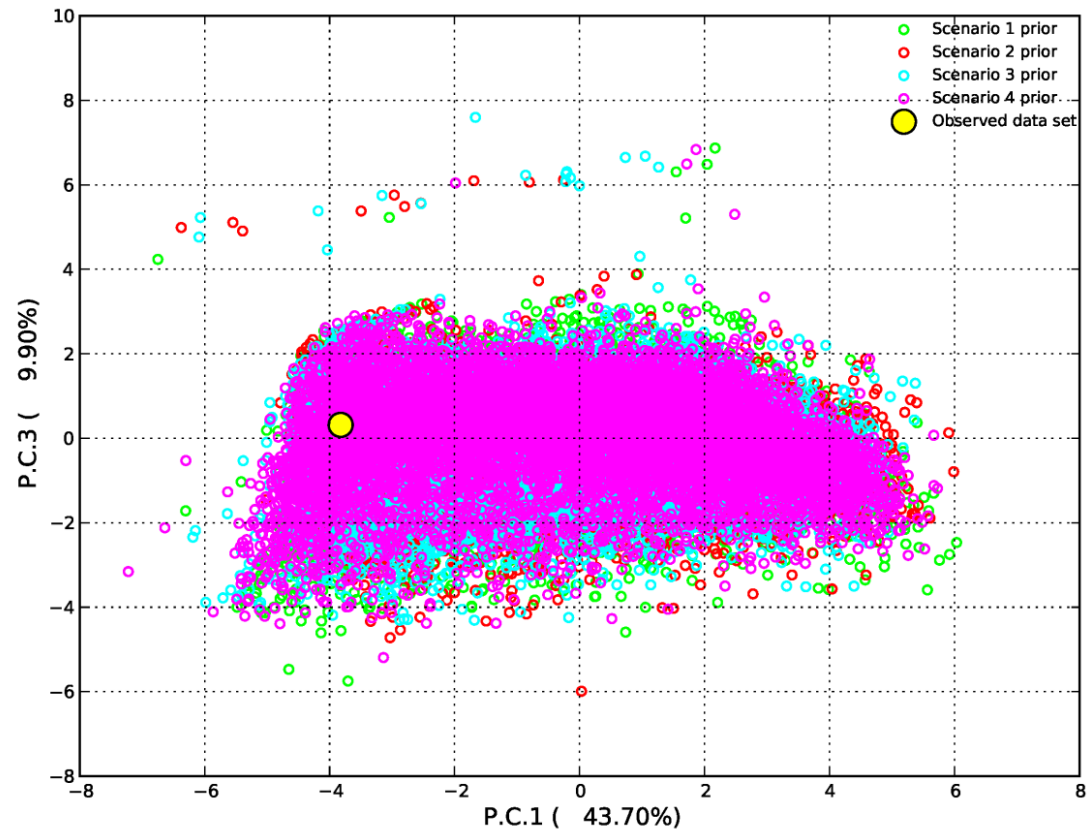

Supplement: Supplementary file 1 — Figure S1 The PCA results of pre‐evaluation of each scenario. Each (small) dot represents a simulated dataset from the reference table and the large yellow dot represents the observed data set. The initial components of datasets are the values of the summary statistics from which the principal components are computed. [file ECE3-12-e9053-s002.pdf]

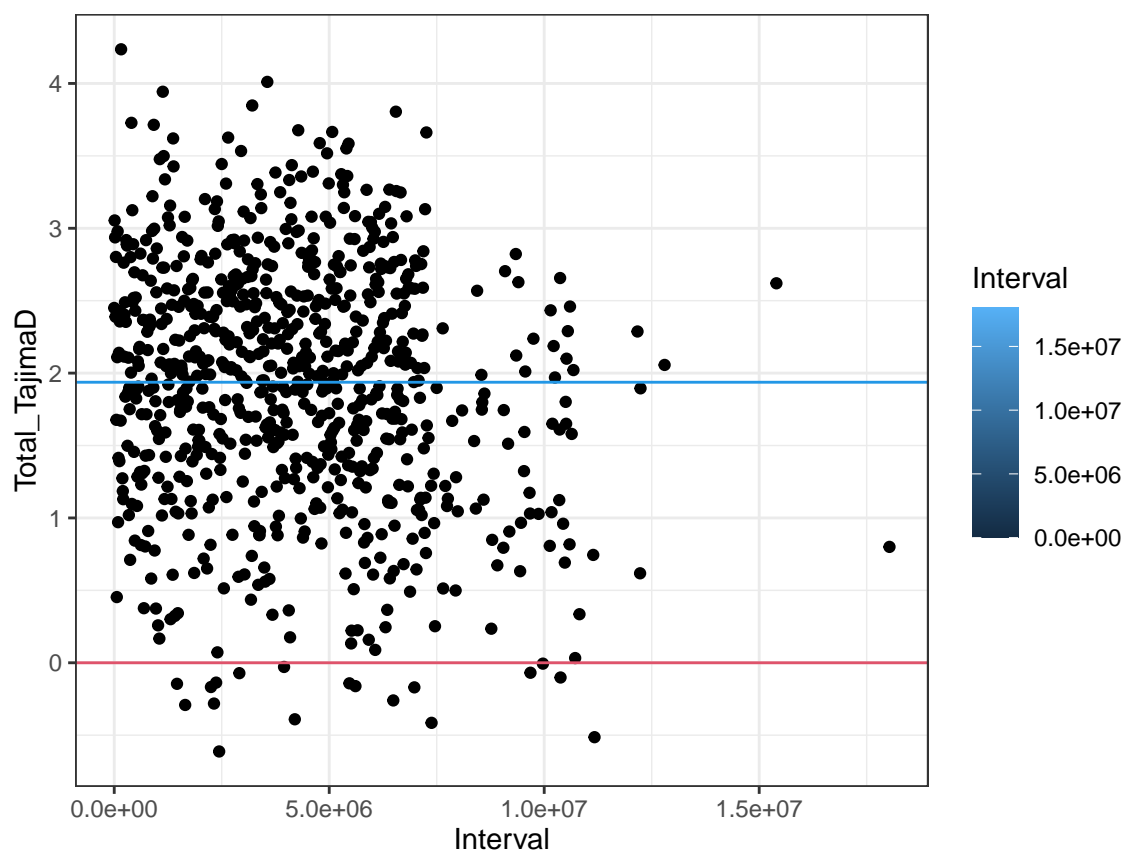

Supplement: Supplementary file 3 — Figure S3 SNPs in the unit sliding window corresponds to Tajima’s D value. Blue line represents the average of Tajima’s D value of SNPs, Tajima’s D = 1.936; Red line represents a dividing line, Tajima’s D = 0 observed variation equal to expected variation. [file ECE3-12-e9053-s001.pdf]

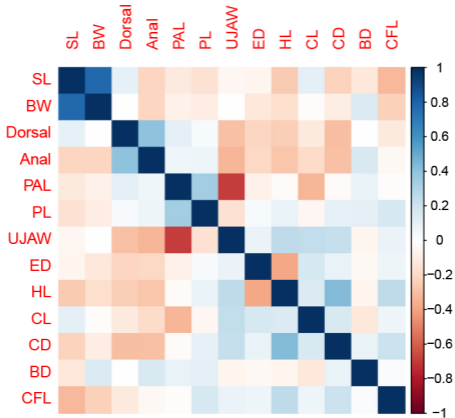

Supplement: Supplementary file 4 — Figure S4 Pearson correlation of 13 standardized phenotypic variables. [file ECE3-12-e9053-s004.pdf]
