# Supplementary material for: ddRAD‐Seq reveals evolutionary insights into population differentiation and the cryptic phylogeography of Hyporhamphus intermedius in Mainland China
Source: Ecol Evol. 2022 Jul 4;12(7):e9053. doi: 10.1002/ece3.9053 (PMC9251877; doi:10.1002/ece3.9053)
Supplement: Supplementary file 5 — Table S1Names of sites sampled, abbreviations of their names, location, and numbers of Hyporhamphus intermedius used in the genomic analyses [file ECE3-12-e9053-s005.docx]

**Table supplements**

Table S1. Names of sites sampled, abbreviations of their names, location and numbers of *Hyporhamphus intermedius* used in the genomic analyses

| Population | Abb | Habitat types | Numbers of samples | Location | Elevation (m) | *Distance (km) | Group |
| --- | --- | --- | --- | --- | --- | --- | --- |
| Danchi | DC | Plateau lake | 20 | 102°45'57.68''E;24°48'46.98''N | 1890 | 1140 | Plateau |
| Fu Xianhu | FXH | Plateau lake | 19 | 102°57'04'' E;24°35'55''N | 1722 | 1110 | Plateau |
| Sanshui | SS | River | 20 | 112°48'45.96''E;23°10'6.04''N | 1 | 120 | Southern |
| Gaoming | GM | River | 19 | 112°51'40.18''E;22°57'48.71''N | 1 | 120 | Southern |
| Doumen | DM | Estuary | 19 | 113° 9'48.06" E; 22°14'34.55" N | 0 | 10 | Southern |
| Chongming | CM | Estuary | 20 | 121°24'22"E;31°36'17.82"N | 0 | 30 | Northern |
| Suzhou | SZ | Lake | 20 | 120°19'34"E;31°16'18.36"N | 3 | 150 | Northern |
| Jining | JN | Lake | 19 | 117°5'2.87"E;34°47'54.38"N | 30 | 180 | Northern |

*Distance: the distance of sites to the nearest estuary

Table S2. Environmental conditions varied among sampling sites.

| Site | WaterT. | Sal. | pH | DO | Chl. | NH4+-N | MD | Air T. | Rainfall | Pressure | DTR | RH |
| --- | --- | --- | --- | --- | --- | --- | --- | --- | --- | --- | --- | --- |
| CM | 19.1 ± 4.56 | 0.2 ± 0.02 | 7.99 ± 0.16 | 7.58 ± 1.2 | 1.5 ± 1.12 | 3.54 ± 1.76 | 30 | 15.8± 8.68 | 94.08 ± 49.66 | 1017.18± 9.5 | 7.4 | 79.58± 3.12 |
| DC | 18.8 ± 4.76 | 0.2 ± 0.01 | 8.7 ± 0.15 | 7.92 ± 1.23 | 11.63 ±3.22 | 1.2 ±0.82 | 1140 | 15.16± 4.47 | 66.65 ± 56.75 | 810.5± 11.3 | 10.58 | 72.08± 8.5 |
| DM | 23.49 ± 5.40 | 0.13 ± 0.03 | 7.56 ± 0.2 | 7.37 ± 1.04 | 3.93 ± 1.22 | 1 ± 0.70 | 30 | 22.46± 5.60 | 188.61 ± 92.32 | 1013.05± 9.12 | 5.85 | 80.08± 7.9 |
| FXH | 17.6 ± 4.12 | 0.24 ± 0.02 | 8.63± 0.12 | 7.79 ± 1.11 | 10.03 ±3.4 | 1.12 ± 0.56 | 1110 | 16.05± 4.38 | 77.09 ± 67.48 | 810± 11.9 | 9.96 | 67.33± 8.33 |
| GM | 23.47 ± 5.12 | 0.12 ± 0.02 | 7.44 ± 0.09 | 7.62 ± 1.32 | 3.6 ± 1.97 | 0.76 ± 0.45 | 113 | 22.2± 5.96 | 139.78 ± 77.82 | 1013.27± 9.2 | 6.7 | 78.58±5.93 |
| JN | 16.49 ± 8.34 | 0.28 ± 0.04 | 8.65 ± 0.12 | 10.54 ± 1.27 | 6.55 ± 2.36 | 1.87 ±0.98 | 180 | 14.35± 10.11 | 55.66 ±54.72 | 1017.27± 8.91 | 10.15 | 67.42±7.43 |
| SS | 23.16 ± 5.23 | 0.12 ± 0.02 | 7.5 ± 0.09 | 7.78 ± 1.34 | 4.86 ± 3.52 | 0.81 ± 0.34 | 139 | 22.18± 5.95 | 140.73 ± 88.06 | 1013.19± 8.7 | 6.9 | 77.67± 5.43 |
| SZ | 17.1 ± 6.36 | 0.28 ± 0.03 | 8.39 ± 0.15 | 8.5 ± 1.05 | 18 ± 7.45 | 1.2 ± 0.45 | 150 | 17.06± 8.81 | 96 ± 45.37 | 1016.7± 8.8 | 7.23 | 73.17± 2.44 |

WaterT.: water temperature (℃)

Sal.: salinity(ppt)

DO: dissolved oxygen(mg/L)

Chl.: chlorophyl a(ug/L)

MD: mouth distance (km)

Air T.: airTemperature(℃)

DTR: daily temperature range

Rainfall (mm)

Pressure (Pa)

RH: relative humidity

Table S3. Effective size in scenario 2 of *Hyporhamphus intermedius* in the DIYABC analyses

| Parameter | mean | median | mode |
| --- | --- | --- | --- |
| ST | 1.17E+03 | 7.17E+02 | 4.46E+02 |
| NT | 8.37E+03 | 8.69E+03 | 9.41E+03 |
| PL | 6.29E+03 | 6.42E+03 | 6.48E+03 |
| t1 | 9.70E+02 | 9.53E+02 | 8.93E+02 |
| t2 | 1.31E+03 | 1.06E+03 | 8.25E+02 |

Number of simulated data sets: 599899

Number of selected data sets: 200000

Table S4. Posterior parameter values for scenario with high probability DIYABC.

|  | Averages Medians | RMedAD |
| --- | --- | --- |
| Scenario 1 |  |  |
| ST | 3.47E+03,5.02E+03 | 0.198 |
| NT | 8.09E+03,5.02E+03 | 0.118 |
| PL | 5.67E+03,5.00E+03 | 0.206 |
| t1 | 5.79E+02,2.96E+03 | 0.243 |
| t2 | 4.06E+03,7.06E+03 | 0.182 |
| Scenario 2 |  |  |
| ST | 1.55E+03,5.02E+03 | 0.455 |
| NT | 8.10E+03,5.00E+03 | 0.13 |
| PL | 6.44E+03,4.99E+03 | 0.196 |
| t1 | 6.22E+02,2.93E+03 | 0.229 |
| t2 | 1.51E+03,7.08E+03 | 0.502 |
| Scenario 3 |  |  |
| ST | 1.42E+03,5.00E+03 | 0.529 |
| NT | 7.09E+03,4.99E+03 | 0.508 |
| PL | 6.15E+03,5.01E+03 | 0.414 |
| t1 | 7.23E+02,2.94E+03 | 0.328 |
| t2 | 1.31E+03,7.07E+03 | 0.722 |
| NA | 7.524e+03,4.999e+03 | 0.181 |
| Scenario 4 |  |  |
| ST | 4.51E+03,5.02E+03 | 0.221 |
| NT | 7.59E+03,5.03E+03 | 0.157 |
| PL | 6.62E+03,5.04E+03 | 0.441 |
| t1 | 2.66E+02,2.95E+03 | 0.33 |
| ra | 2.49E,01,2.51E,01 | 0.114 |
| t2 | 4.77E+03,7.11E+03 | 0.207 |

RMedAD : the Relative Median Absolute Deviation

Table S5. Redundancy analysis summary statistics of genotype-phenotype association

|  | RDA1 | RDA2 | RDA3 | RDA4 | RDA5 | RDA6 | RDA7 | RDA8 | RDA9 | RDA10 |
| --- | --- | --- | --- | --- | --- | --- | --- | --- | --- | --- |
| Eigenvalues | 1234.469 | 119.32255 | 79.6233 | 39.243 | 27.09735 | 25.8989 | 23.87951 | 21.73806 | 20.85612 | 16.41698 |
| Proportion Explained | 0.7674 | 0.07418 | 0.0495 | 0.0244 | 0.01685 | 0.0161 | 0.01485 | 0.01351 | 0.01297 | 0.01021 |
| Cumulative Proportion | 0.7674 | 0.84163 | 0.8911 | 0.9155 | 0.93237 | 0.9485 | 0.96331 | 0.97683 | 0.98979 | 1 |
| environmental variables | p-value | r^2^ | Vif.cca | |  |  |  |  |  |  |
| SL | 0.001 | 0.1559 | 1.430 | |  |  |  |  |  |  |
| Dorsal | 0.001 | 0.4046 | 1.359 | |  |  |  |  |  |  |
| Anal | 0.001 | 0.7269 | 1.725 | |  |  |  |  |  |  |
| PL | 0.012 | 0.0539 | 1.097 | |  |  |  |  |  |  |
| UJAW | 0.001 | 0.1445 | 1.290 | |  |  |  |  |  |  |
| ED | 0.001 | 0.056 | 1.751 | |  |  |  |  |  |  |
| HL | 0.002 | 0.0703 | 2.234 | |  |  |  |  |  |  |
| CL | 0.001 | 0.2032 | 1.202 | |  |  |  |  |  |  |
| CD | 0.001 | 0.1812 | 1.478 | |  |  |  |  |  |  |
| BD | 0.004 | 0.0758 | 1.090 | |  |  |  |  |  |  |
| *R^2^_adj_* | 0.2438 |  |  | |  |  |  |  |  |  |
